# Effect of Bacterial and Yeast Starters on the Formation of Volatile and Organic Acid Compounds in Coffee Beans and Selection of Flavors Markers Precursors During Wet Fermentation

**DOI:** 10.3389/fmicb.2019.01287

**Published:** 2019-06-26

**Authors:** Silvia Juliana Martinez, Ana Paula Pereira Bressani, Disney Ribeiro Dias, João Batista Pavesi Simão, Rosane Freitas Schwan

**Affiliations:** ^1^Department of Biology, Federal University of Lavras, Lavras, Brazil; ^2^Department of Food Science, Federal University of Lavras, Lavras, Brazil; ^3^Technology and Coffee Growing Course, Federal Institute of Espírito Santo, Alegre, Brazil

**Keywords:** coffee quality, cells adhesion, organic acids, volatile compounds, starters culture

## Abstract

Coffee quality has recently become a high demand of coffee consumers, due to all the specialty coffees available on the market. Specialty coffees can be generated by favoring growth of some groups of microorganisms during fermentation or by using starters. Just as yeast, a variety of bacteria can be used to generate important flavor precursors. The aim of this work was to test the efficiency of coffee sterilization and adhesion of microbial cells on beans, to evaluate the effect of yeast and bacterial starters on the production of organic and volatile compounds, and selection of potential flavor marker precursors during the wet fermentation. Three yeast and six bacterial starters were inoculated in coffee beans. Coffee sterilization and microbial adhesion was observed by scanning electron microscopy (SEM). Organic compounds were detected by high performance liquid chromatography (HPLC) and volatile compounds by gas chromatography–mass spectrometry (GC–MS). Micrographs from the SEM showed that sterilization was efficient, because there were no microbial cells after autoclaving for 5 min. Also, it was observed an increase of microbial cells from 0 to 48 h of fermentation. Malic, lactic, and acetic acid were only detected in the bacterial treatments. Volatile compounds: 4-ethenyl-1,2-dimethoxybenzene, heptadecanol, 4-hydroxy-2-methylacetophenone, and 1-butanol,2-methyl were only found in yeast treatments. Guaiacol was only produced by the inoculated *B. subtilis* starters. In conclusion, yeast starters were better producers of volatile alcohols and bacterial starters of acid compounds. This study allowed the selection of potential flavor marker precursors, such as heptadecanol, 4-hydroxy-2-methylacetophenone, 7-methyl-4-octanol, and guaiacol.

## Introduction

Coffee quality has recently become a high demand of coffee consumers and is directly related to its chemical compounds, especially those that constitute flavor. This important sensorial attribute of coffee is produced by several volatile compounds that are generated before and mainly during roasting. Moreover, they are responsible for the generation of specialty coffees. However, the chemical and volatile profiles vary and are dependent of environmental factors, altitude, coffee variety, storage conditions (Evangelista et al., 2014a), processing methods, and degree of roasting (Hurtado-Benavides et al., 2016).

The processing method is an important phase which involves fermentation. Three processing methods are mainly used: dry, semi-dry (also called honey or pulped natural), and wet (Silva, 2015). In the wet method coffee fruits are first depulped mechanically, leaving part of the mucilage. Then, beans are put in fermentation tanks with water, allowing fermentation take place for 6 to 72 h (depending on the environmental temperature), followed by drying (Avallone et al., 2001; Evangelista et al., 2015). Coffee from the wet method contains a higher acidity than the other methods (Peñuela-Martínez et al., 2018). Acidity is due to the lowering of the pH by bacteria during wet fermentation.

Many studies have characterized spontaneous fermentation, from which strains have been isolated and use as starters. Yeasts starters are the most studied and use to ferment coffee, due to their favorable contributions of flavor in the beverage (Evangelista et al., 2014a,b; Martinez et al., 2017; Bressani et al., 2018). Some of those flavor attributes are: caramel, chocolate, herbaceous materials, yellow fruits, and almonds. In addition, yeast starters reduce the processing time and drying, improving the sensory quality and economic value of the product (Silva et al., 2013; Evangelista et al., 2014a). Yeast can also inhibit mycotoxigenic fungal growth, as demonstrated by Souza et al. (2017). Few works implementing bacterial starters in coffee have been conducted, most of them use lactic acid bacteria (LAB) such as Wang et al. (2018).

Since characterization of the microbial groups and chemical compounds during fermentation using starters have been continuously done; there are no works on the adhesion of microbial cells from starters to the surface of beans. Techniques such as SEM can offer that information. The SEM can scan surfaces, crystalline structures, chemical compositions, and electron behaviors of samples (Vernon-parry, 2000). In coffee, the SEM was used to observe the endosperm of coffee beans and the integrity of cell walls and membranes in the final stage of drying with varied temperatures in natural and depulped coffee (Sondahl and Baumann, 2001; Borém et al., 2013). Since SEM does not provide enough information to have an overall understanding of the effect of starters on coffee beans, other analyses are needed, for example chromatographic techniques. Using chromatography, sugars, organic acids, and volatiles can be evaluated, and specially can assist on the selection of important makers flavor precursors that characterizes better the processes and starters.

The present work aimed to test the efficiency of coffee sterilization and to assess the adhesion of microbial cells to beans by scanning electron microscopy. Moreover, this study aimed to evaluate using refined techniques the effect of yeast and bacterial starters on the production of organic acids and volatile compounds in order to select potential flavor marker precursors during the wet fermentation of coffee.

## Materials and Methods

### Coffee Variety

Ripe fruits of *Coffea arabica* cv Catuaí Vermelho were harvested manually on a farm that was 750–800 m above sea level located in Lavras, state of Minas Gerais, Brazil. The fruits were processed using the wet method. The mature fruits were collected, washed, and separated by density. Then, they were depulped and part of their mucilage was removed mechanically. The resulted beans were later used to start the wet fermentation, inside containers filled with water.

### Coffee Starter Cultures

The yeasts and bacteria isolated from different varieties and processes of coffee (Table 1) carried out in Minas Gerais were obtained from the Culture Collection of Agricultural Microbiology (CCMA, Federal University of Lavras, Lavras, Minas Gerais, Brazil).

**Table 1:** Detailed information for the starter origins and features.

ID^∗^	Microorganism	Isolation source		Features	Literature
		Process	Coffee variety		
CCMA 0543	*Saccharomyces cerevisiae*	Dry and Semidry	Acaiá	High pectin enzymatic activity. Presence of desirable compounds.	Silva et al., 2000, 2013; Vilela et al., 2010.
CCMA 0544	*Candida parapsilosis*				
CCMA 0684	*Torulaspora delbrueckii*				
CCMA 1203	*Pantoea dispersa*	Wet	Ouro Amarelo, Mundo Novo and Catuaí Vermelho	High citric acid production and volatiles.	NYP
CCMA 1236	*Bacillus subtilis*			High pectinolytic enzymatic activity.	
CCMA 1238	*Bacillus subtilis*				
CCMA 1239	*Bacillus subtilis*				
CCMA 1242	*Bacillus subtilis*				
CCMA 1279	*Arthrobacter koreensis*			Have pectinolytic enzymatic activity.	

^∗^*ID from the Microbiology Agriculture Culture Collection. NYD, not yet published*.

The yeast strains were reactivated in 10 mL of Yeast Extract Peptone Glucose (YEPG) broth (20 g/L glucose, 10 g/L yeast extract, and 10 g/L soya peptone) and bacterial strains in 10 mL nutrient broth, with incubations at 28°C/48 h for yeast and 30°C/48 h for bacteria. Then, yeast strains were transferred to 50 mL YEPG and bacterial strains to 50 mL nutrient broth and incubated at 28 and 30°C with agitation of 120 rpm/24 h. The yeast cells were transferred to larger volumes of YEPG until they reached a concentration of 10^9^ cells/mL, and the same was done for bacterial cells using nutrient broth. Subsequently, the cells were centrifuged and resuspended in distilled water (enough for inoculation without increasing the moisture of the coffee).

### Sterilization Conditions of Coffee Beans Samples and Inoculation

Before inoculation, matured beans were sterilized in glass containers and in an autoclave for 5 min 121°C/15 psi. Autoclaved beans were implemented in this work to ensure that the compounds detected were originated from the starter’s strains, and to see whether 5 min of sterilization were enough to unable contaminant microorganism’s growth.

After, 150 g of sterilized water was added into the glass containers that had 150 g of autoclaved coffee beans and inoculated by yeast and bacterial suspensions. Each strain was inoculated separately in triplicate. Consequently, there were three yeast strains, six bacteria strains, and one control that had no microbial inoculation. Seven grams of samples were collected after 0, 24, and 48 h of fermentation and placed in sterile plastic bags for further analyses.

### Study of the Sterilization and Adhesion of Microbial Cells by SEM

#### Sample Preparation

Autoclaved coffee beans from control and with inoculation were analyzed under the microscope. The preparation and observation of samples under a scanning electron microscope was performed at the Laboratory of Electron Microscopy and Ultra Structural Analysis (LME), located in the Department of Plant pathology/UFLA.

The samples were cut longitudinally and immersed in fixative solution (modified Karnovsky: 2.5% glutaraldehyde, 2.5% formaldehyde in 0.05 M sodium cacodylate buffer, pH 7.2, 0.001 M CaCl2) and stored for 1 week in a cold chamber until analysis. Then, the obtained samples were washed with cacodylate buffer (3 times, leaving each buffer 10 min) inside the exhaust hood to remove the residues. After removing the cacodylate buffer from the last wash, the samples were dehydrated with increasing concentrations of acetone (25, 50, 75, 90, and 100%, the solution of 100% should be used three times) for approximately 10 min each. Subsequently, the samples were transferred to the critical point apparatus to complete the drying. The dried samples were then placed on aluminum stubs covered with aluminum foils with carbon strips placed on top. After assembly, sputtering in a gold bath was applied followed by microscopic observation.

#### SEM Analysis

The stubs with gold were placed on the SEM LEO EVO 40 PVX. The microscope was turned on, and the images were digitally generated and recorded. The images were observed with increasing variables, using a working condition of 20 kv and a working distance of 9 mm. The generated images were recorded and opened in Corel Draw Software package, where they were selected and prepared.

### Chemical Analyses

#### Organic Acids by High-Performance Liquid Chromatography (HPLC)

Organic acids of coffee beans were evaluated after 0, 24 and 48 h of fermentation. The extractions and operating conditions were performed as described by Evangelista et al. (2014b). Three grams of coffee beans was used for analysis. Each sample was homogenized in Falcon tubes with 20 mL of 16 mM perchloric acid and Milli-Q water by vortexing at room temperature for 10 min. The extracts were centrifuged at 12745 RCF for 10 min at 4°C. The pH of the supernatant was adjusted to 2.11 using perchloric acid and recentrifuged under the same conditions. The second supernatant was filtered through a 0.22 μm cellulose acetate membrane and directly injected (20 μL) onto the chromatographic column.

Later, samples were analyzed using a high performance liquid chromatography (HPLC) system (Shimadzu Corp., Japan) equipped with a detection system consisting of a UV–Vis detector (SPD 10Ai) and a Shimpack SCR-101H (7.9 mm × 30 cm) column operating at 50°C, which was used to achieve chromatographic separation of water-soluble acids that were eluted with 16 mM of perchloric acid at a flow rate of 0.6 mL/min. The acids were identified by comparison with retention times of authentic standards. The quantification was performed using calibration curves constructed with standard compounds [malic and citric acid were purchased from Merck (Germany), lactic and tartaric acid were purchased from Sigma-Chemical (EUA), acetic and succinic acids were purchased from Sigma-Aldrich (Germany), isobutyric and isovaleric acid were purchased from Riedel-deHaen (Germany)].

#### Volatile Compounds

Volatile compounds were extracted from samples of green coffee (0, 24, and 48 h of fermentation) using headspace-solid phase microextraction (HS-SPME) according to Evangelista et al. (2014b). The compounds were analyzed using a Shimadzu QP2010 GC model equipped with mass spectrometry (MS) and a silica capillary Carbo-Wax 20M (30 m × 0.25 mm × 0.25 mm) column. The operating conditions were performed as described by Bressani et al. (2018). The volatile compounds were identified by comparing the mass spectra to the NIST11 library. In addition, an alkane series (C10–C40) was used to calculate the retention index (RI) for each compound and compared with the RI values found in the literature data (Czerny and Grosch, 2000; Eom and Jung, 2013; Piccino et al., 2014).

### Statistical Analysis

The concentrations of organic compounds were evaluated by a normality test, that helped deciding if the statistical test that needs to be apply is parametric or not. Then, the means were analyzed using the XLSTAT software (Excel-Addinsoft, New York, NY, United States) by the Friedman’s test at *p* < 0.05. Data of Volatile compounds were statistically analyzed by principal component analysis (PCA) using Chemoface software (Nunes, 2016). In which a m × n matrix was built with the relative areas, were *n* was the value of each identified peak of volatile compound and m the different treatments of strains plus control.

## Results and Discussion

### Yeast and Bacterial Strains in Coffee

All microorganisms used for inoculation were isolated from different coffee fermentation processes. These strains were dominant microorganisms from coffee fermentation. The employed yeast strains have been previously inoculated and monitored by Silva et al. (2013), Evangelista et al. (2014a), Martinez et al. (2017), and Bressani et al. (2018). *Candida parapsilosis* is one of the fungi most frequently isolated from human hands, is considered a killer yeast and fungal antagonist based on its ability to produce chemicals that exert cytotoxic effects on the cells of other organisms (Robledo-Leal et al., 2014). Candida species are also reported to lyse Ca2+-pectate. Similarly, yeasts can produce enzymes degrading polysaccharides of the mucilage.

Regarding the strains of bacteria, this work was the first to use these strains isolated from previous works on coffee done in State of Minas Gerais. The *Bacillus* genus is known to be found during the whole wet fermentation process (Silva, 2015). *B. subtilis* possesses pectinases, xylanases and enzymes depolymerising polysaccharides. It is a useful strain to be used as starter. Genus *Pantoea* is well known as a phytopathogen of plants and fruits. However, some Pantoea strain works as plant growth promoting microorganism and in biocontrol. This is the first description of the specie *Pantoea dispersa* in coffee. Finally, the *Arthrobacter* genus is found in the rhizosphere of plants (Álvarez-Rodríguez et al., 2003).

### Efficiency of Sterilization and Adhesion of Microbial Cells

**Figure 1:**
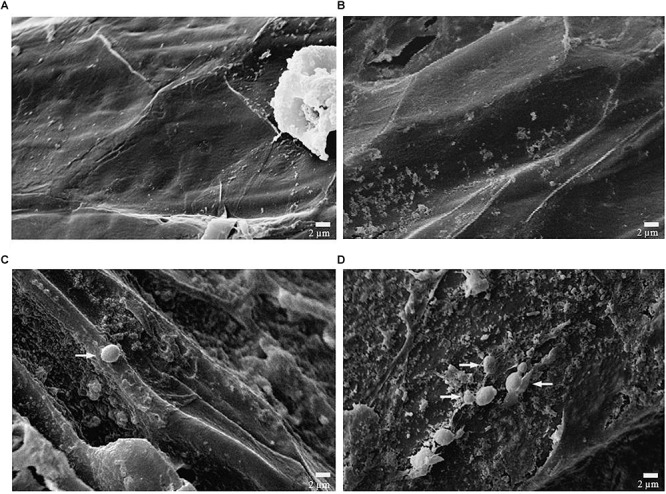
Scanningelectron micrographs of coffee beans from control and inoculated samples with CCMA 0543 processed by the wet method. **(A-top left)** Surface of control bean at 0 h, **(B-top right)** surface of control bean after 48 h of fermentation, **(C-bottom left)** surface of inoculated bean at 0 h (after inoculation), and **(D-bottom right)** surface of inoculated bean after 48 h of fermentation.

The scanning electron micrographs shown in Figure 1 demonstrates the efficiency of sterilizing coffee beans and adhesion of inoculated microbial cells from strain CCMA 0543 in comparison to the control during wet fermentation. As shown in Figure 1A,B, there was no microbial growth after 0 or 48 h of fermentation, which was expected since it was the control. Meaning that 5 min was enough to inhibit epiphytic microbial growth. As described by Ibrahim et al. (2011), autoclaving involves high pressure and temperature that allows boiling of the substrate and denaturation of proteins, which leads to killing of the microorganisms (Madigan et al., 2012).

Figure 1C,D shows inoculated coffee beans after 0 and 48 h of fermentation. Figure 1C shows that microbial cells were not present in higher quantities as in Figure 1D, probably due to the adaptation phase they must go through before multiplying. Figure 1D also demonstrates the oval shape and budding reproduction of yeast cells. At 48 h, the micrograph shows that microbial cells are adhered to the bean mucilage, probably due to the carbon compounds available, that would be used for their growth (Silva, 2015).

Although fewer cells than anticipated were adhered to the mucilage of the bean, the other cells may have remained in the fermented liquid, not being able to adhere. The wet method uses depulped coffee submersed in water for fermentation, and therefore the fermented liquid is part of it. Before fermentation, cherries in the wet process go through mechanical removal of pulp and a large part of the mucilage (Evangelista et al., 2015), which might possibly affect the concentration of cells that can adhere to the mucilage when compared to the dry and semidry method.

### Starter Effect on Organic Acid Compounds

**Figure 2:**
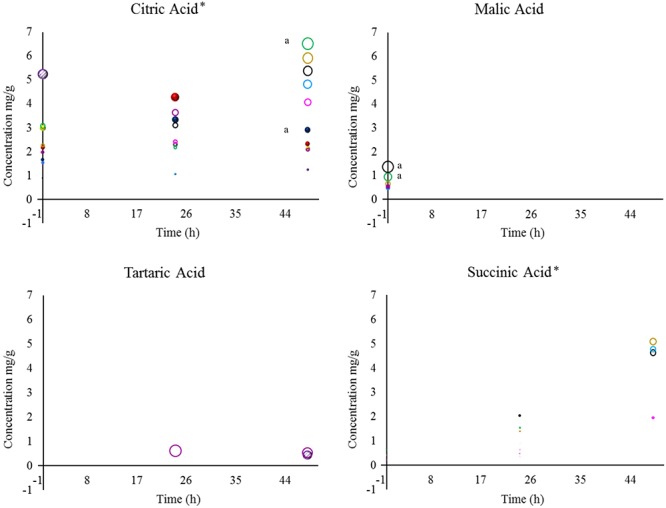
Concentrations of citric, malic, tartaric, and succinic acid detected in coffee beans during wet fermentation.Inoculation with *S. cerevisiae* CCMA 0543 (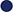), *C. parapsilosis* CCMA 0544 (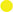), *T. delbrueckii* CCMA 0684 (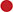), *Pantoea dispersa* CCMA 1203 (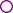), *Bacillus subtilis* CCMA 1236 (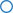), *Bacillus subtili* CCMA 1238 (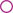), *B. subtilis* CCMA 1239 (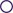), *B. subtilis* CCMA 1242 (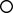), *Arthrobacter koreensis* CCMA1279 (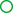), and control (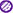). Compounds with the symbol “^∗^” were the most significant when compared to all treatments, based on their mean values, at *p* < 0.05 by Friedman’s test. When analyzing each compound, a lowercase “a” was designated for means values of treatments that were the most significant at *p* < 0.05 by Friedman’s test.

**Figure 3:**
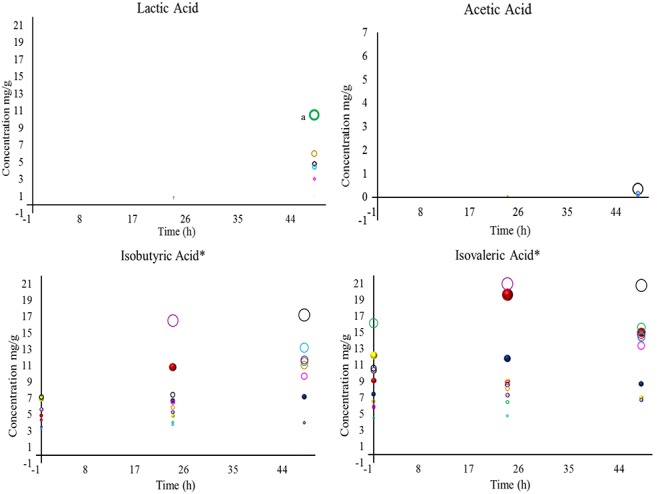
Concentrations of lactic, acetic, isobutyric, and isovaleric acid detected in coffee beans during wet fermentation. Inoculation with *S. cerevisiae* CCMA 0543 (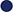), *C. parapsilosis* CCMA 0544 (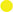), *T. delbrueckii* CCMA 0684 (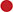), *Pantoea dispersa* CCMA 1203 (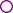), *Bacillus subtilis* CCMA 1236 (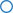), *Bacillus subtilis* CCMA 1238 (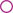), *Bacillus subtilis* CCMA 1239 (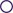), *Bacillus subtilis* CCMA 1242 (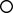), *Arthrobacter koreensis* CCMA 1279 (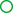), and control (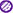). Compounds with the symbol “^∗^” were the most significant when compared to all treatments, based on their mean values, at *p* < 0.05 by Friedman’s test. When analyzing each compound, a lowercase “a”; was designated for means values of treatments that were the most significant at *p* < 0.05 by Friedman’s test.

Natural organic acids are the most versatile ingredients in the food and flavor industry. These compounds have the following three main properties: they are soluble in alcohols, water or other solvents, hygroscopic, and they have buffering and chelation abilities (Daneshfar et al., 2012; Schaft, 2015; Vandenberghe et al., 2018). The organic acids that were evaluated during wet fermentation are shown in Figure 2, 3.

In total eight acids were detected. When analyzing statistically all obtained acids from yeast and bacterial treatments, the presence and concentration of citric, succinic, isovaleric, and isobutyric acid were the most significant (acids marked with ^∗^ in Figure 2, 3). Although citric acid and malic acid were expected to be significant because they are always the most predominant acids in green coffee, other acids also interfere with the sensory characteristics of coffee by affecting sweet flavors and acidity (Oestreich-Janzen, 2013; Ribeiro et al., 2017).

During fermentation, the production of undesirable acids, such as butyric and propionic acid, were not detected in this study, which is a good sign since they confer off-flavors such as onion to the beverage and showed that the beans were not over-fermented (Bade-Wegner et al., 1997; Silva, 2015; Haile and Kang, 2019). Even though is believed that species of *Bacillus* genus are responsible for the production of propionic acid (Silva, 2015), none of the *Bacillus* stains used in this work produced this acid. A study describing the wet fermentation also did not detect these undesirable acids (Evangelista et al., 2015).

One of the properties that can be conferred by citric acid is tartness, fruity, and berry flavors (Vandenberghe et al., 2018). In this work, within yeast treatments, only CCMA 0543 showed a significant difference in citric acid at the end of fermentation (2.90 mg/g). This result can be use by producers if they intend of having fruity notes in their coffee. When comparing all strains and control, only treatment with CCMA 1279 had a significant concentration of citric acid at the end of fermentation (6.52 mg/g). Being both concentrations are within the standard values of Arabica coffee in the wet process (Peñuela-Martínez et al., 2018). As in Evangelista et al. (2015), in our work it was observed a decrease in citric acid for control. Treatment with starter CCMA 1242 showed the highest amount of isovaleric acid at the end of fermentation.

The control had no presence of malic, succinic, lactic, and acetic acid. Malic, lactic, and acetic acid were only detected in treatments with bacteria, since they can be related to their metabolic activity (Avallone et al., 2001). Succinic acid was detected for all bacterial starters and yeast starters CCMA 0544 and 0684, which is a good indicator when using yeast inoculation. Moreover, succinic acid is a main organic acid produced by yeasts and is formed in the glyoxylate cycle by oxidation of isocitrate as well as in the reductive citric acid cycle (Jayaram et al., 2014). Apart from succinic acid, citric, isobutyric, and isovaleric acid were also detected in yeast starters, with the difference that they were also present in the bacterial starters and control. It can be inferred that since citric, isobutyric and isovaleric acid were also present in the control (with no inoculation), either they are naturally derived acids that were products of reactions taking place in coffee fruits, such as the Krebs cycle (Vandenberghe et al., 2018), or pre-fermentation occurred immediately after the fruits were torn from the coffee plants.

Tartaric acid was present in the control after 48 h of fermentation (0.43 mg/g) and in bacterial treatment CCMA 1203 from 24 h (0.61 mg/g) to 48 h of fermentation (0.54 mg/g). This finding indicated that since is in control, tartaric acid may probably be a naturally derived acid from coffee fruits and that with inoculation its production could be stimulated earlier and enhance fruit flavor such as grape (Dziezak, 2016). The production of acetic acid was observed in treatments with CCMA 1242, 1239, 1238, and 1236 after 48 h of fermentation, except for treatment with CCMA 1239, in which it started after 24 h. Lactic acid was detected after 24 h in treatments with CCMA 1279, 1239, 1238, and 1203, and after 48 h in treatments with CCMA 1236 and 1242; a significant concentration remained in the treatment of CCMA 1279. For the production of acetic and lactic acid, the bacteria in this work might have fermented the sugars available or used citric acid as a source, as recently recorded in a similar fermentation such as cocoa fermentation (Ho et al., 2018). Malic acid was only present at the start of fermentation for treatments with CCMA 1279, 1242, 1239, 1236, and 1203, demonstrating the highest and significant concentrations in CCMA 1279 and 1242.

### Effects on Volatile Compounds and the Selection of Potential Markers

**Figure 4:**
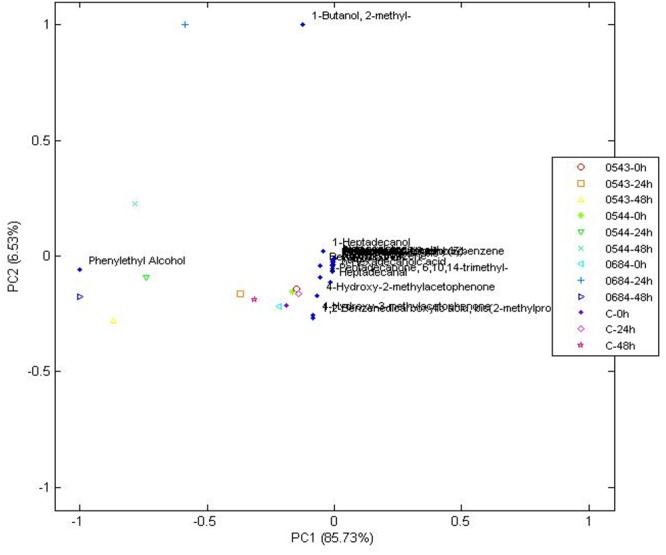
Principal components analysis of volatile compounds in yeasts when compared to the control at 0, 24, and 48 h, during wet fermentation.

Yeast exhibit different metabolic processes and secrete different types of extracellular enzymes during fermentation, suggesting that fermentation of green coffee beans with suitable yeast species would modify the flavor precursors and volatile profiles (Lee et al., 2017). This suggestion has not been deeply explored for bacterial starters, because there are a few reports involving bacteria starters and only LAB was used (Wang et al., 2018). The volatiles identified after 0, 24, and 48 h of wet fermentation for every treatment and control are illustrated on Table 2 and their PCA is on Figure 4, 5. From the 24 compounds illustrated in Table 2, there were 5 alcohol, 4 acids, 3 ketones, 3 esters, 2 phenols, 1 aromatic, 1 aldehyde, 1 pyrrole, 1 furan, and 3 other compounds, and not all of them were present in all treatments or the control.

**Table 2:** Total volatile compounds for each starter during wet fermentation. Mean values of areas ± SD.

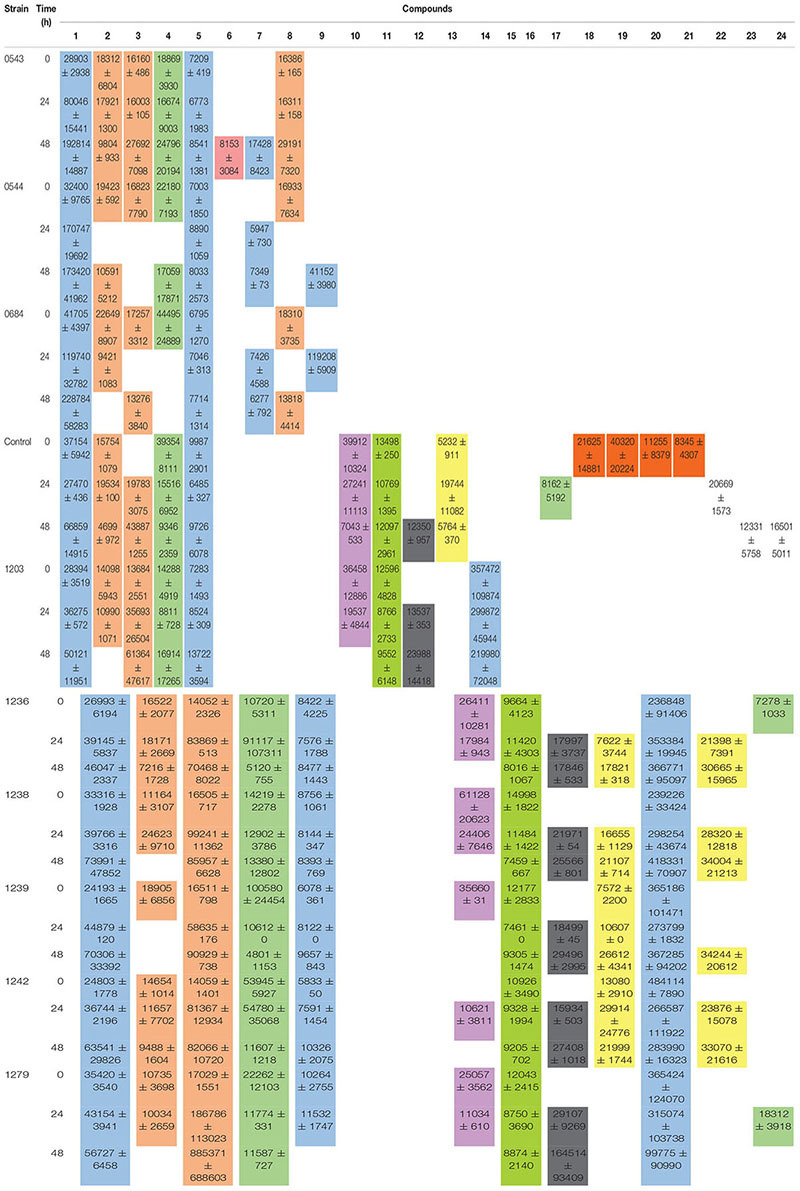

*Every color indicates a different group of compounds: **—** alcohol, **—** ketone, **—** ester, **—** aromatic, **—** aldehyde, **—** pyrrole, **—** furan, **—** phenol, and **—** acid. 1 = Phenylethyl Alcohol; 2 = 2-Pentadecanone, 6,10,14-trimethyl-; 3 = 4-Hydroxy-3-methylacetophenone; 4 = 1,2-Benzenedicarboxylic acid, bis(2-methylpropyl) ester; 5 = Benzyl alcohol; 6 = 4-Ethenyl-1,2-dimethoxybenzene; 7 = 1-Heptadecanol; 8 = 4-Hydroxy-2-methylacetophenone; 9 = 1-Butanol, 2-methyl-; 10 = Heptadecanal; 11 = Indole; 12 = Benzofuran, 2,3-dihydro-; 13 = Hydroxybenzene; 14 = 7-Methyl-4-octanol; 15 = Guaiacol; 16 = Methyl salicylate; 17 = Homosalate; 18 = Myristic acid; 19 = n-Hexadecanoic acid; 20 = 9-Octadecenoic acid (Z)-; 21 = Pentadecanoic acid; 22 = Octane, 1,1′-oxybis-; 23 = Dimethyl sulfide; 24 = Heneicosane*.

**Figure 5:**
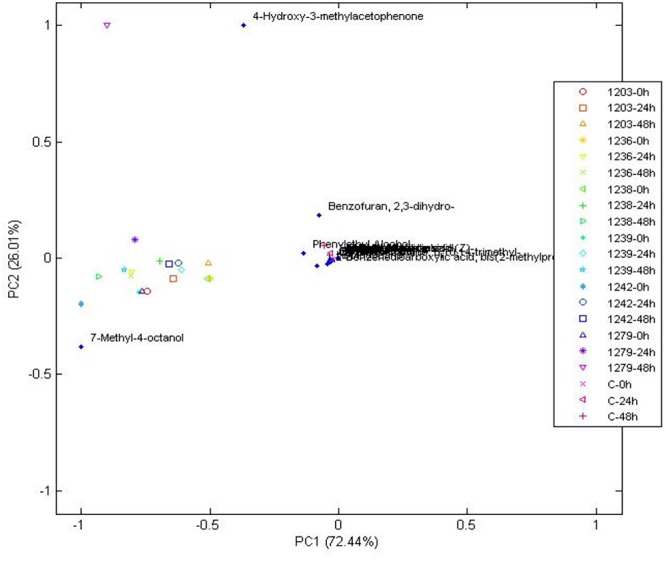
Principal components analysis of volatile compounds in bacteria when compared to the control at 0, 24, and 48 h, during wet fermentation.

Phenethyl alcohol is a higher alcohol that produces rose-like flavors and is generated during phenylalanine metabolism mainly by yeasts (Fukuda et al., 1990; Verhagen, 2010; Liu, 2015; Michel et al., 2016). Phenethyl alcohol was detected in control, all yeast and bacterial treatments. Furthermore, the PCA on Figure 4 showed that phenethyl alcohol better characterized yeast treatments with CCMA 0543, 0544, and 0684 at 48 h when compared with the control. Due to presence of Phenethyl alcohol in the control, which means that it could have been generated after the beans were collected from the plants, it cannot be used as a potential marker since it was not produced only by the starters. However, it can be used as a key flavor marker to indicate that microorganisms are metabolically active.

The compound that better characterized most bacterial treatments based on the PCA was 7-methyl-4-octanol (Figure 5). This compound was only detected in bacterial treatments at all times of fermentation when compared to the control and yeast treatments (see Table 2). It is one of the compounds that can be used as a potential marker to indicate the presence of bacteria during a wet coffee fermentation. Information about this compound is limited. In a cereal-based fermented food, 7-methyl-4-octanol is an important compound produced by the *Lactobacillus plantarum* strain (Ferri et al., 2016).

There were other compounds that were only found in yeast treatments when compared to the control or bacterial treatments, such as 4-ethenyl-1,2-dimethoxybenzene (also known as styrene), heptadecanol, 4-hydroxy-2-methylacetophenone, and 1-butanol,2-methyl (Table 2). Although their presence was not steady in the times of fermentation evaluated, they can be used as potential flavor marker precursors in green beans when inoculating coffee with yeasts in a wet process, especially with the starters *S. cerevisiae* CCMA 0543, *C. parapsilosis* CCMA 0544, and *T. delbrueckii* CCMA 0684.

Heptadecanol and 4-hydroxy-2-methylacetophenone were detected in the three yeast species starters in comparison to 1-butanol,2-methyl (CCMA 0544 and CCMA 0684) and 4-ethenyl-1,2-dimethoxybenzene (CCMA 0543). Heptadecanol is part of the volatile alcohol compounds produced by yeasts, particularly from the species *S. cerevisiae* as shown by Utto et al. (2012), who isolated yeasts from fermented soybean curd and tested their volatile production. The ketone 4-hydroxy-2-methylacetophenone confers important properties to coffee: anticancer and antioxidant activities (Dandekar et al., 2015). The 4-ethenyl-1,2-dimethoxybenzene was only present in the treatment with *S. cerevisiae* CCMA 0543 at the end of fermentation, which is in accordance with Gramatica et al. (1981) and Bhulya et al. (2015), who observed the production of 4-ethenyl-1,2-dimethoxybenzene by decarboxylation of cinnamic acids using *S. cerevisiae*. Another property of this compound in green beans, in addition to be an important flavor constituent, is its antioxidant activity (Arfmann and Abraham, 1989). Finally, 1-butanol,2-methyl is a higher alcohol that is generated from 2-keto acids via the last two steps of the Ehrlich pathway in yeasts (Vogt et al., 2016). In the present study, this alcohol was only found in treatments with CCMA 0544 and 0684.

Other compounds that were only found in bacterial treatments were guaiacol (also known as 2-methoxyphenol) and ester methyl salicylate. As in yeast, these two compounds can be used as potential flavor marker precursors in green beans for some of the species used as starters. Guaiacol is produced through the non-oxidative decarboxylation of vanillic acid by several bacteria such as *Bacillus megaterium* and *B. subtilis*, among others (Álvarez-Rodríguez et al., 2003; Witthuhn et al., 2012). In accordance with the literature, our work showed that guaiacol was only produced by the inoculated *B. subtilis* starters (see Table 2), which indicates that it can be used as a strong marker precursor for the presence of this species during fermentation. Although esters can be present at low concentrations, their production can highly influence coffee flavor by generating fruity flavors. In the beer industry, this group represents the largest and most important group of active flavor compounds (Dack et al., 2017). They are mainly produced by acid catalysis and a decrease in pH, and they are mostly derived from yeasts (Ramey and Ough, 1980; Kumazawa, 2006; Garavaglia et al., 2014). As observed in our work, it is possible that bacteria contribute to esterification of methyl salicylate from the beans. Mainly because the ester compound methyl salicylate was only detected in the bacterial strains CCMA 1236 (at the beginning) and CCMA 1279 (24 h). Methyl salicylate is synthesized from salicylic acid, which is a plant hormone, its produced as a plant defense from microorganisms, and in the beverage generates flavor attributes such as warm, sweet, mint-like, and rooty-fruity (Flament, 2002; Kalaivani et al., 2016).

## Conclusion

The scanning electron micrographs demonstrated that surface sterilization of coffee beans was efficient for the elimination of microorganisms, making it an excellent method to test strains in the generation of important flavor compounds. Additionally, microbial cells adhered to the surface of bean mucilage during wet fermentation. The use of yeast starters was better for volatile alcohol production, and bacterial starters were better for acid production, because, although both have important characteristics for beverages, each strain provides a particular characteristic. A group that could be characterized as important in this work consisted of the esters, one of was produced only by bacterial strains CCMA 1236 and CCMA 1279. During analysis, this study allowed the selection of potential flavor marker precursors through the wet fermentation of green beans. For yeast, heptadecanol and 4-hydroxy-2-methylacetophenone can be used as markers, and for bacteria in general, 7-methyl-4-octanol can be used to ascertain their presence and metabolically active status during fermentation, whereas guaiacol can be used for *B. subtilis* species.

## Data Availability

The raw data supporting the conclusions of this manuscript will be made available by the authors, without undue reservation, to any qualified researcher.

## Author Contributions

RS conceived the study. RS, DD, SM, and AB designed the experiments. SM and AB performed the experiments. All authors interpreted the results and performed the data analysis. RS and DD contributed reagents, materials, and analysis tools. SM wrote the manuscript. All authors read, reviewed, and approved the final manuscript.

## Conflict of Interest Statement

The authors declare that the research was conducted in the absence of any commercial or financial relationships that could be construed as a potential conflict of interest.
